# Dietary Zinc Supplementation Improves Growth, Antioxidant Capacity, Immunity, and Intestinal Health in Juvenile Black Carp (*Mylopharyngodon piceus*)

**DOI:** 10.3390/biology15120939

**Published:** 2026-06-16

**Authors:** Jiaxing Yu, Penghui Zhang, Xunshang Zhang, Xiaotong Zhu, Yuanyuan Xie, Hao Zhang, Xianping Shao, Mingxu Xie, Yan Liu, Xia Yang, Chenglong Wu

**Affiliations:** School of Life Science, Huzhou Normal University, 759 East 2nd Road, Huzhou 313000, China; y15615528313@outlook.com (J.Y.); penghuizhang2021@outlook.com (P.Z.); xunshangzhang@outlook.com (X.Z.); xiaotongzhu2026@outlook.com (X.Z.); xieyuanyuan23@outlook.com (Y.X.); zhlyyxxf@outlook.com (H.Z.); shaoxp@zjhu.edu.cn (X.S.); 03247@zjhu.edu.cn (M.X.); 02870@zjhu.edu.cn (Y.L.); yangxia@zjhu.edu.cn (X.Y.)

**Keywords:** *Mylopharyngodon piceus*, Zinc, growth performance, antioxidant capacity, immunity and inflammatory responses

## Abstract

Zinc (Zn) is an essential trace element that plays important roles in growth, digestion, antioxidant defense, immunity, and inflammation regulation in fish. This study investigated the effects of graded dietary Zn levels on growth performance, serum biochemistry, digestive enzyme activity, zinc transporter expression, antioxidant capacity, immune responses, and inflammatory regulation in juvenile black carp (*Mylopharyngodon piceus*). Six isonitrogenous and isoenergetic diets were formulated to contain 27.95, 34.38, 44.90, 66.52, 116.14, and 199.56 mg/kg Zn by supplementing ZnSO_4_·7H_2_O. Juvenile fish with an initial weight of 2.88 ± 0.12 g were fed the experimental diets for 60 days in triplicate tanks. Growth performance increased with dietary Zn and then plateaued at 44.90–199.56 mg/kg; broken-line regression estimated the optimal dietary Zn requirement at 44.6 mg/kg. Adequate Zn supplementation also reduced whole-body lipid content, increased digestive enzyme activities, improved serum HDL-C and ALP levels, and decreased AST and ALT activities. In addition, adequate dietary Zn (44.90 mg/kg) significantly modulated the expression of zinc transporter genes in the liver and intestine. Adequate dietary Zn supplementation enhanced antioxidant capacity by activating the Nrf2/Keap1 signaling pathway, improved intestinal immunity, and strengthened barrier function by increasing the expression of tight junction proteins and mucins. Moreover, adequate dietary Zn could alleviate inflammatory responses by upregulating anti-inflammatory factors and downregulating pro-inflammatory cytokines via the *MAPK14* signaling pathway. These findings suggest that dietary zinc at 44.60 mg/kg is sufficient to promote growth, antioxidant status, immune function, and intestinal health in juvenile black carp.

## Simple Summary

Zinc is an essential trace mineral that plays important roles in fish growth, digestion, antioxidant defense, immunity, and inflammation regulation. However, the zinc requirement of juvenile black carp (*Mylopharyngodon piceus*) has not been clearly defined. In this study, juvenile black carp were fed diets containing graded levels of zinc for 60 days to evaluate growth performance, serum biochemical indices, digestive enzyme activities, zinc transport, antioxidant responses, intestinal immunity, inflammatory responses, and intestinal barrier function. The results showed that adequate dietary zinc significantly improved growth and feed efficiency, with an optimal dietary zinc requirement of approximately 44.6 mg/kg. Appropriate zinc supplementation also enhanced digestive enzyme activity, promoted zinc transporter expression, activated the antioxidant pathway via Nrf2/Keap1 pathway, improved intestinal immune defenses, strengthened intestinal barrier integrity, and suppressed inflammation through the *MAPK14* signaling pathway. These findings provide a scientific basis for optimizing dietary zinc supplementation in juvenile black carp feed formulations.

## 1. Introduction

As an essential trace element, zinc (Zn) acts as a crucial cofactor for over 300 enzymes, playing an indispensable role in sustaining growth performance and physiological functions in both terrestrial animals and teleosts [1]. Zn deficiency adversely affects various species by retarding growth, impairing metabolic functions, and compromising both antioxidant capacity and immunity [2,3]. At the molecular level, Zn uptake is facilitated by specific transporters, which are vital for maintaining intracellular homeostasis and regulating metabolic processes [4,5]. The systemic and cellular Zn levels are meticulously regulated by Zn transporter (ZnT) families, which mediate Zn flux across biological membranes to preserve systemic balance [6,7,8]. Consequently, determining precise dietary Zn requirements is fundamental to ensuring metabolic homeostasis and optimal growth across diverse species.

In teleost fish, maintaining Zn homeostasis is critical for physiological equilibrium and aquaculture success. Although fish can absorb trace amounts of waterborne Zn via their gills, this is rarely sufficient to meet the high metabolic demands of rapidly growing cultured species, making a precise dietary supply imperative [9]. Disruption of this balance—either through deficiency or excessive accumulation—severely impairs fish health, leading to growth retardation, metabolic dysfunction, or heavy metal toxicity [10]. Metabolically, adequate dietary Zn significantly enhances nutrient assimilation by upregulating the activities of key digestive enzymes, such as amylase, trypsin, and lipase, in the hepatopancreas and intestine [11,12]. Furthermore, intensive aquaculture often exposes fish to environmental stressors, triggering the overproduction of reactive oxygen species (ROS) and subsequent oxidative stress. In response, Zn serves a pivotal function in the antioxidant defense system [13]. It protects against oxidative damage and tissue injury by activating the Nrf2 signaling pathway, which upregulates downstream target molecules and antioxidant enzymes, including catalase, heme oxygenase-1 (HO-1), and glutathione [11,12], a protective mechanism widely conserved across diverse species [14,15,16,17].

Beyond its metabolic and antioxidant functions, Zn is also fundamental to maintaining intestinal health, immunomodulation, and the suppression of inflammation. Intestinal health is intrinsically dependent on structural integrity. Extensive research demonstrates that optimal dietary Zn fortifies the intestinal barrier by upregulating tight junction proteins, such as occludin and claudins [18,19]. Concurrently, Zn enhances host intestinal immune defenses by stimulating the activity of immunomodulatory factors, including lysozyme (LZM) and complement components 3 and 4 (C3, C4) [20]. Furthermore, Zn mitigates intestinal inflammation by optimizing the cytokine profile. Specifically, it suppresses the expression of pro-inflammatory mediators, such as tumor necrosis factor-α (TNF-α) and interleukin-1β (IL-1β), while promoting the secretion of anti-inflammatory cytokines, such as interleukin-10 (IL-10) and transforming growth factor-β1 (TGF-β1) [20,21,22]. Mechanistically, this immunomodulation is largely achieved through the regulation of inflammatory signaling cascades. Pro-inflammatory stimuli frequently activate the p38 MAPK signaling pathway, which governs vital cellular processes including differentiation and apoptosis [11,12]. Numerous studies have evidenced that sufficient dietary Zn inhibits the overactivation of the p38 MAPK pathway, thereby curbing the excessive production of inflammatory factors and alleviating inflammation-induced tissue damage in the intestine [22,23].

Black carp (*Mylopharyngodon piceus*) is a commercially vital freshwater carnivorous teleost widely cultured in China, playing a crucial role in the aquaculture industry. Although recent studies have explored its nutritional requirements for amino acids [24], vitamins [20,25], and certain minerals [26], the precise dietary Zn requirement for this species remains completely undefined. Given its unique molluscivorous feeding habits and the intensive nature of its culture, extrapolating Zn requirements from other cyprinid species may lead to inaccurate feed formulations, potentially resulting in suboptimal growth, impaired Zn homeostasis, or compromised immunity. Therefore, establishing the optimal dietary Zn inclusion level specifically for *M. piceus* is highly necessary. The specific objectives of this study were to determine the precise dietary Zn requirement for juvenile black carp based on growth performance and systematically evaluate the impacts of graded dietary Zn levels on serum biochemistry, Zn transporter gene expression, hepato-intestinal antioxidant status, and intestinal immune and inflammatory responses. Ultimately, these findings will provide a crucial scientific basis for optimizing Zn supplementation in practical aquafeeds, thereby enhancing the health, growth, and economic sustainability of black carp aquaculture.

## 2. Materials and Methods

### 2.1. Animals, Diets and Treatments

Six isonitrogenous and isoenergetic experimental diets were formulated to evaluate the dietary Zn requirements of juvenile black carp (*M*. *piceus*) (Table 1). Casein and gelatin were used as the primary protein sources, fish oil as the lipid source, and dextrin as the carbohydrate source. To strictly control the basal Zn level, a vitamin premix and a Zn-free mineral premix were added to all diets. Zinc sulfate heptahydrate (ZnSO_4_·7H_2_O) was supplemented into the basal diet at graded levels of 0, 10, 20, 40, 80, and 160 mg/kg (designated as Zn0, Zn10, Zn20, Zn40, Zn80, and Zn160). Although no exogenous zinc was supplemented in the mineral premix, a basal level of zinc was still present in the control diet, which was attributed to the endogenous Zn contained in the dietary protein sources.

According to the Inductively Coupled Plasma Mass Spectrometry (ICP-MS) analysis, the actual dietary Zn concentrations were 27.95, 34.38, 44.90, 66.52, 116.14, and 199.56 mg/kg, respectively. The basal Zn content (27.95 mg/kg) in the unsupplemented control group (Zn0) originated purely from the inherent background Zn present in the raw dietary ingredients. All dry ingredients were finely ground, sieved, thoroughly mixed with oil and water, and extruded into 2.0 mm pellets. The pellets were air-dried and stored at −20 °C until further use. The production and storage protocols strictly followed the guidelines described by Jia et al. [25].

### 2.2. Fish and Feeding Trial

All experimental procedures received prior approval from the Ethics Committee of Huzhou Normal University (Protocol No. 068/2023) and adhered to standard protocols for animal welfare. The experimental procedures in this study were adapted from those previously described by Wu et al. [20] and Jia et al. [25]. Healthy black carp (*M. piceus*) were bought from Xingwang Fish Breeding Farm (Huzhou, China).

Before the experiment, all fish were acclimated in 500 L tanks for two weeks and fed the control diet (Zn0). Subsequently, 540 fish of uniform size (initial body weight: 2.88 ± 0.12 g) were randomly distributed into 18 tanks (30 fish per tank), representing six dietary treatments with three biological replicates (tanks) per treatment. The feeding trial lasted for 60 days. Fish were hand-fed to apparent satiation (approximately 4% of body weight) three times daily (08:00, 12:00, and 17:00). To accurately calculate the feed conversion ratio (FCR), uneaten feed residues were siphoned out one hour after each feeding, oven-dried, and weighed. Feces were removed daily. Strict water quality management was maintained throughout the trial with a daily water exchange rate of approximately 25%. The water quality parameters were monitored daily and maintained within the following ranges: water temperature at 26.0 ± 1.0 °C, dissolved oxygen (DO) > 6.0 mg/L, pH at 7.6 ± 0.1, and total ammonia nitrogen < 0.1 mg/L. The experiment was conducted in an indoor flow-through system with a natural photoperiod.

### 2.3. Sample Collection and Compositional Analysis

At the end of the feeding trial, all fish were fasted for 24 h to empty their digestive tracts. Following anesthesia with tricaine methanesulfonate [27], all fish from each tank were counted and batch-weighed to evaluate growth performance. Subsequently, 10 fish were randomly sampled from each tank and pooled together to form one composite sample per tank, representing one biological replicate (*n* = 3 biological replicates per dietary treatment). Serum, liver, and intestine samples were immediately harvested on ice, flash-frozen in liquid nitrogen, and stored at −80 °C. The proximate compositions of the experimental diets and whole fish bodies (moisture, crude protein, crude lipid, and ash) were analyzed following standard AOAC methods as described in our previous studies [20,25].

### 2.4. Biochemical and Enzymatic Assays

Serum samples (*n* = 3 biological replicates per group, each pooled from 10 fish) were analyzed for the levels of albumin (ALB), total bile acid (TBA), glucose (GLU), and the activities of aspartate aminotransferase (AST), alkaline phosphatase (ALP), lactate dehydrogenase (LDH), and alanine aminotransferase (ALT). These indices were measured using an automated clinical chemistry analyzer (Model C400n, Dirui, Changchun, China).

For digestive enzyme analysis, frozen liver and intestinal tissues were pulverized in liquid nitrogen and homogenized in a 9-fold volume of ice-cold phosphate-buffered saline (PBS, pH 7.4). The homogenates were centrifuged at 3000× *g* for 20 min at 4 °C, and the supernatants were collected. The activities of lipase (LPS), amylase (AMS), trypsin (TRY), and chymotrypsin (CYT) were determined using commercial assay kits (Nanjing Jiancheng Bioengineering Institute, Nanjing, China) with three technical replicates per sample.

### 2.5. Analysis of Antioxidative, Oxidative, Immune, and Inflammatory Markers

The hepatic and intestinal antioxidant statuses were evaluated by determining the activities of superoxide dismutase (SOD), catalase (CAT), glutathione reductase (GR), glutathione S-transferase (GST), glutathione peroxidase (GPX), as well as the levels of glutathione (GSH), total antioxidant capacity (T-AOC), and malondialdehyde (MDA). Intestinal immune parameters, including lysozyme (LZM), immunoglobulin M (IgM), acid phosphatase (ACP), and alkaline phosphatase (ALP), were also assessed. All biochemical analyses were conducted using commercial kits (Nanjing Jiancheng, Bioengineering Institute, Nanjing, China) strictly according to the manufacturer’s instructions.

Furthermore, the intestinal concentrations of complement components (C3, C4) and inflammatory cytokines (TGF-β1, TNF-α, IL-1β, IL-6, IL-8, IL-12, IL-17, and IFN-γ) were quantified using specific Fish ELISA kits (Hengyuan Biotech, Shanghai, China). For all biochemical and ELISA analyses, samples from *n* = 3 tanks per treatment were utilized to ensure true biological replication.

For histological analysis, fixed intestine samples (4% paraformaldehyde) were dehydrated, embedded in paraffin, and sectioned at 5 μm thickness. Sections were stained with hematoxylin and eosin (H&E) and examined under a light microscope (Olympus BX53, Tokyo, Japan) to evaluate morphological integrity. Specifically, morphometric parameters including villus height, villus width, muscular thickness, and crypt depth were quantified using cellSens Standard software version 1.18 (Olympus, Tokyo, Japan). For each section, at least 5 intact and well-oriented villi were randomly selected to measure and calculate the average values.

### 2.6. Analysis of Gene Expression

Total RNA from hepatic and intestinal tissues (*n* = 3 biological replicates per group) was extracted using the TRIzol reagent (Invitrogen, Carlsbad, CA, USA). First-strand cDNA was synthesized using the MonScript RT-PCR Kit (Monad Biotech, Wuhan, China). Specific primer sequences (Table 2) were synthesized by Biosune Biotech (Shanghai, China). Quantitative real-time PCR (qPCR) was performed in triplicate (technical replicates) on a CFX96 Real-Time PCR System (Bio-Rad, Hercules, CA, USA) using SYBR Green Master Mix (Takara, Dalian, China). Thermal cycling conditions followed Wu et al. [20], and relative mRNA expression was calculated via the 2^−ΔΔCt^ method normalized to β-actin.

### 2.7. Data Analysis

All data are presented as means ± standard deviations (SDs). Statistical analyses were performed using IBM SPSS software version 25.0 (Chicago, IL, USA). The normality and homogeneity of variance were verified prior to analysis. Differences among dietary treatments were evaluated using one-way ANOVA, followed by Duncan’s multiple range test. Orthogonal polynomial contrasts were utilized to determine linear and quadratic dose–response trends. A difference was considered statistically significant at *p* < 0.05. The optimal dietary Zn requirement was estimated using a broken-line regression model based on weight gain and specific growth rate.

## 3. Results

### 3.1. Growth Performance and Body Composition

**Figure 1:**
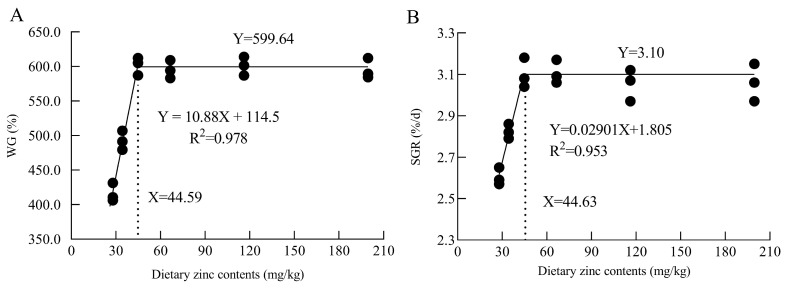
Estimation of the optimal dietary zinc requirement for juvenile black carp using broken-line regression analysis. (**A**) weight gain (WG) showing an optimal level of 44.59 mg/kg, and (**B**) specific growth rate (SGR) showing an optimal level of 44.63 mg/kg.

Relative to the unsupplemented control (Zn0), dietary zinc inclusion significantly stimulated FBW, WG, and SGR (*p* < 0.05) (Table 3). Notably, WG and SGR reached their maxima in the Zn20 group. Based on broken-line regression models applied to WG and SGR, the optimal zinc requirements for juvenile black carp (*M. piceus*) were calculated as 44.59 and 44.63 mg/kg, respectively (Figure 1). Furthermore, zinc addition (Zn10–Zn160) led to a marked reduction in the FCR in comparison with the deficient group (*p* < 0.05); no statistical variations were presented among these supplemented treatments (*p* > 0.05). HSI and CF remained statistically unaffected by dietary treatments (*p* > 0.05).

Regarding proximate composition, moderate to high dietary zinc (Zn20–Zn160) significantly suppressed whole-body crude lipid content relative to the Zn0 and Zn10 groups (*p* < 0.05). However, whole-body protein and ash fractions exhibited no significant responsiveness to zinc levels (*p* > 0.05).

### 3.2. Biochemical Parameters in the Serum

HDL-C level was significantly increased and then maintained a stable trend in the serum of the Zn20, Zn40, Zn80 and Zn160 groups in comparison with the Zn-deficient group (Zn0) (*p* < 0.05). Conversely, Zn supplementation (Zn10–Zn80) significantly decreased serum LDL-C, GLU, and AST levels relative to the Zn0 control (*p* < 0.05), with these parameters plateauing and showing no further statistical variation among the Zn20 to Zn80 treatments (*p* > 0.05). Although there were no significant variations between the Zn0 and Zn160 treatments (*p* > 0.05), ALT activities were notably decreased in the serum of the Zn10, Zn20, Zn40 and Zn80 groups in comparison with the Zn0 group (*p* < 0.05). However, there were no variations in the contents of TG, TC, ALB and TBA among these six treatments (*p* < 0.05) (Table 4 and Table 5).

### 3.3. Expression of Zn Transport-Related Genes

**Figure 2:**
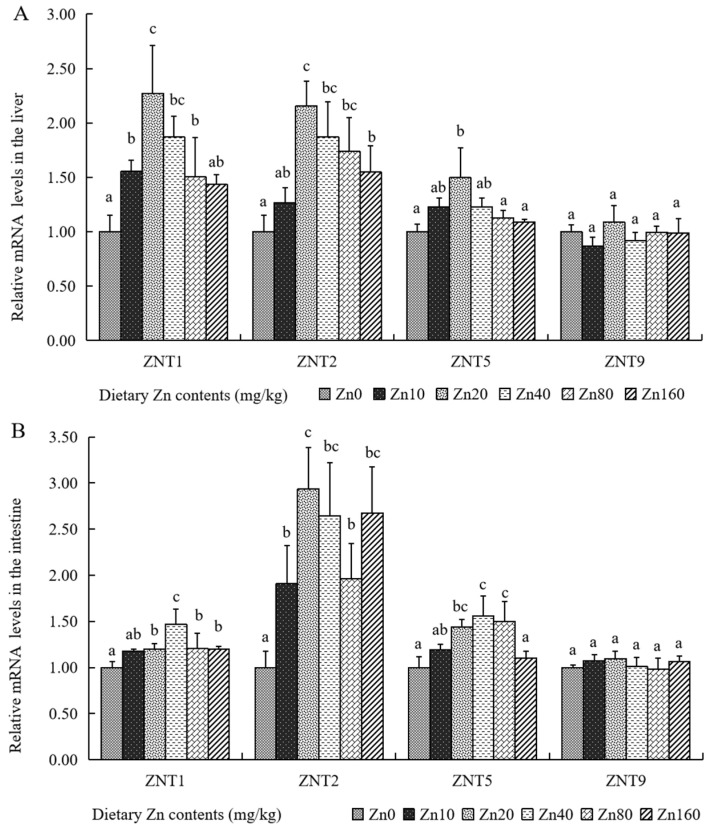
Effect of different levels of zinc on expression levels of Zn transport-related genes in juvenile black carp. (**A**) Expression levels in the liver; (**B**) Expression levels in the intestine. Significant differences (*p* < 0.05) among groups are indicated by different letters above the bars.

Transcriptional profiles of zinc transporters in targeted tissues are illustrated in Figure 2. In the liver, *ZNT1*, *ZNT2*, and *ZNT5* transcript abundances peaked within the Zn20 cohort (*p* < 0.05) before declining at higher dietary zinc doses. Although hepatic *ZNT2* remained elevated in the Zn160 treatment compared to the deficient control, *ZNT1* and *ZNT5* expressions returned to baseline, showing statistical equivalence to the Zn0 group (*p* > 0.05). Regarding intestinal tissues, ZN*T1* mRNA levels were initially upregulated, reaching maximal level in the Zn40 group (*p* < 0.05), followed by a marked suppression under the Zn80 and Zn160 diets (*p* < 0.05). Similarly, intestinal *ZNT2* expression in the Zn40 cohort surged to approximately threefold that of the unsupplemented control. Notably, *ZNT9* transcription exhibited no significant responsiveness to dietary zinc variations in either the liver or intestine across all treatments.

### 3.4. Hepatic Antioxidative and Oxidative Parameters

**Figure 3:**
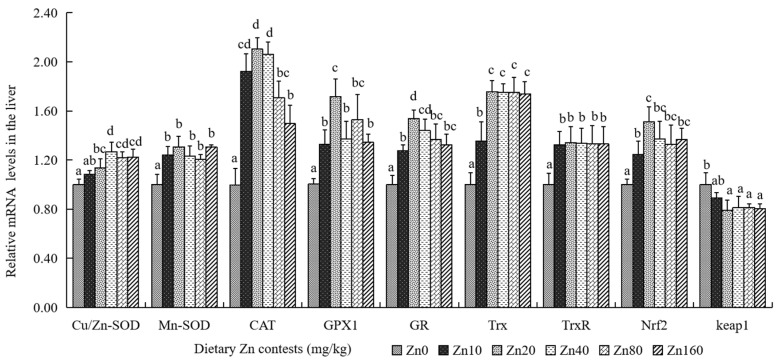
Effect of different levels of zinc on expression levels of antioxidant-related genes in the liver of juvenile black carp. Different lowercase letters above the bars indicate significant differences among the treatments (*p* < 0.05).

As outlined in Table 6, dietary zinc inclusion significantly stimulated hepatic antioxidant defenses. Specifically, the activities of T-SOD, CAT, GPX, and GR, together with GSH and T-AOC levels, all peaked in the Zn20 treatment (*p* < 0.05). Conversely, GST activities and MDA contents were significantly reduced in Zn-supplemented groups compared to the control, indicating decreased oxidative stress (*p* < 0.05). Dietary Zn levels significantly affected the hepatic mRNA expression of antioxidant-related genes in juvenile black carp (*M. piceus*) (*p* < 0.05) (Figure 3). Overall, Zn supplementation upregulated the expression of Cu/Zn-SOD, Mn-SOD, GR, CAT, GPX1 and Nrf2 to varying extents, whereas Keap1 displayed an opposite trend.

Specifically, *Cu/Zn-SOD* increased progressively with Zn inclusion and reached the highest level at the Zn40 trial group (*p* < 0.05), and there were no significant differences from the 40 mg/kg group (*p* > 0.05) but remained significantly higher than the Zn0 and Zn10 groups (*p* < 0.05). *Mn-SOD* was notably elevated in all Zn-supplemented groups compared to the Zn-deficient group (*p* < 0.05), showing an overall increase followed by maintenance. *CAT* was the most Zn-responsive gene: it was markedly enhanced at Zn10 to Zn80 groups and peaked at Zn20 (*p* < 0.05), followed by a significant decline at Zn160 (*p* < 0.05), though still higher than the Zn0 group (*p* < 0.05). *GPX1* was significantly increased and maximized at Zn20 (*p* < 0.05); it then decreased at higher Zn levels (40–160 mg/kg) but generally remained above the Zn0 level (*p* < 0.05). *GR* peaked at 20 mg/kg (*p* < 0.05), with the Zn40 mg/kg group ranking second, and then declined further at Zn80 and Zn160 mg/kg (*p* < 0.05), suggesting an optimal response window. Regarding transcriptional regulators, *Nrf2* was significantly upregulated by Zn and reached the maximum at Zn20 (*p* < 0.05), while the Zn40–Zn160 treatments maintained relatively high but lower-than-peak levels. In contrast, *Keap1* was significantly reduced at Zn10 compared with Zn0 (*p* < 0.05) and decreased further at Zn20 to Zn160, remaining at the lower levels with no marked variations among high-Zn groups (*p* > 0.05).

Collectively, antioxidant-related genes presented a pronounced non-linear dose–response to dietary Zn. Stronger induction was generally observed within the Zn20 to Zn40 range (with *Cu/Zn-SOD* favoring 40 mg/kg, whereas *CAT*, *GPX1*, *GR* and *Nrf2* were more prominent at 20 mg/kg), while partial attenuation or plateauing occurred at higher Zn levels. The coordinated increase in *Nrf2* and decrease in *Keap1* further suggest that Zn supplementation may facilitate hepatic antioxidant transcriptional regulation.

### 3.5. Intestinal Antioxidant Capacity and Oxidative Status

As summarized in Table 7, dietary zinc inclusion upregulated intestinal T-SOD, CAT, GPX, and GR activities, as well as GSH levels, which all culminated in the Zn20 treatment. Significant elevations in T-AOC were also observed from the Zn20 group onwards (*p* < 0.05), maintaining stability at higher zinc doses (*p* > 0.05). In contrast, increasing zinc intake triggered an initial reduction in GST activity, hitting the lowest point in the Zn40 cohort (*p* < 0.05).

**Figure 4:**
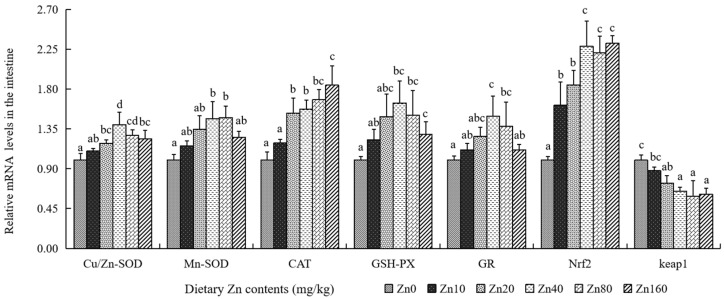
Effect of different levels zinc on expression levels of antioxidant-related genes in the intestine of juvenile black carp. Significant differences (*p* < 0.05) among groups are indicated by different letters above the bars.

Dietary zinc significantly altered the transcriptional profiles of intestinal antioxidant genes (Figure 4). In comparison with the Zn-deficient diet, dietary Zn administration uniformly upregulated the mRNA transcriptinal levels of *Cu/Zn-SOD*, *Mn-SOD*, *CAT*, *GSH-PX*, and *GR* (*p* < 0.05). Specifically, the mRNA levels of *Cu/Zn-SOD*, *Mn-SOD*, and *GR* were heightened and achieved their peak levels in the Zn40 treatment, followed by a declining trend at higher doses. Meanwhile, the transcription amounts of *CAT* were positively correlated with dietary Zn contents and achieved their maximal levels in the Zn160 treatments (*p* < 0.05). Specifically, the highest transcriptional abundance of *GSH-PX* was recorded in the Zn80 treatment (*p* < 0.05). In addition, the transcription levels of the key regulator, *Nrf2*, were markedly elevated and reached a plateau in the Zn40 to Zn160 groups. In contrast, the transcription amounts of its inhibitor, *Keap1*, were notably reduced in the Zn-supplemented groups, with the lowest levels observed from the Zn40 to Zn160 treatments (*p* < 0.05).

### 3.6. Intestinal Immunity and Inflammatory Responses

Compared with the zinc-deficient group, zinc administration significantly bolstered gut immunity (*p* < 0.05). Notably, both C3 and IgM contents peaked across the Zn10 and Zn20 groups. Lysozyme (LZM) reached its highest concentration in groups Zn20 and Zn40, while C4 was maximized in the Zn20 and Zn80 groups. Furthermore, enzyme activities were also affected. ACP activity was greatest in the Zn20 group, whereas ALP activity exhibited a progressive, dose-dependent increase, culminating in the Zn160 group (*p* < 0.05).

Compared with the Zn-deficient group (Zn0), the transcription levels of complement-related genes (*C3*, *C4* and *CFB*) were markedly heightened in the Zn-supplemented groups (*p* < 0.05). Specifically, *C3* was markedly increased and achieved higher levels in Zn40-Zn80 treatments compared with Zn0 treatment, followed by a significant decrease in the Zn160-treated group (*p* < 0.05), although it remained higher than Zn0 (*p* < 0.05). Relative to the Zn0 treatment, *C4* concentrations were significantly augmented from Zn10 to Zn40 (*p* < 0.05), thereafter reaching a stable plateau with no marked variances across the Zn20–Zn160 treatments (*p* > 0.05). Similarly, *CFB* contents experienced a sharp enhancement that peaked at Zn40, stabilizing subsequently at values persistently greater than the unsupplemented group (*p* < 0.05).

**Figure 5:**
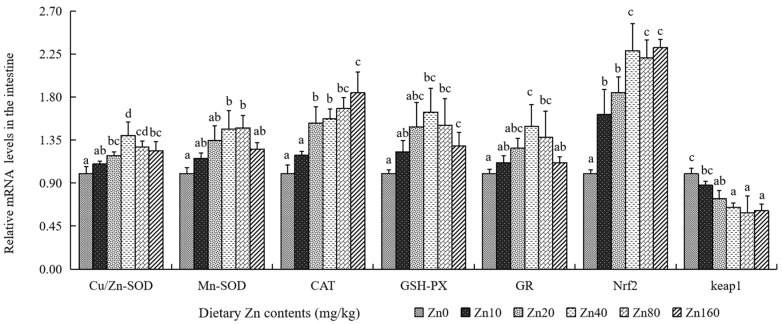
Effect of different levels of dietary zinc on the relative mRNA expression of immune-related genes in the intestine of juvenile black carp. Significant differences (*p* < 0.05) among groups are indicated by different letters above the bars.

Regarding intestinal gene expression (Figure 5), the transcriptional abundances of *LZM*, *NRAMP*, and *IgH* shared a highly consistent parabolic trend. They were maximally upregulated in the Zn40 cohort (*p* < 0.05) before undergoing a marked decline under excessive Zn inputs (Zn80 and Zn160), although their final expressions still generally surpassed the Zn-deficient baseline (*p* < 0.05). Conversely, *Igλ1* transcription displayed a milder sensitivity to Zn inclusion; its expression was primarily enriched in the Zn40 and Zn80 treatments compared to the lower-dosage groups, exhibiting statistical equivalence among the three highest Zn levels (Table 8).

### 3.7. Analysis of Intestinal Inflammatory and Anti-Inflammatory Parameters

At the protein level (Table 9), dietary Zn inclusion significantly boosted the concentrations of classic anti-inflammatory cytokines (TGF-β1 and IL-10) (*p* < 0.05). Conversely, a pronounced suppression was observed in the pro-inflammatory cascade molecules (IL-1β, IL-6, TNF-α, and IFN-γ) across all supplemented cohorts, where the minima were recorded in the Zn20 and Zn40 treatments (*p* < 0.05).

**Figure 6:**
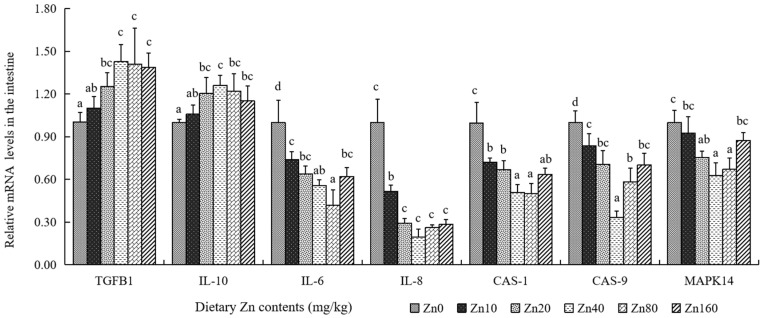
Effect of different levels of zinc on expression levels of anti-inflammatory and inflammatory responses’ genes in the intestine of juvenile black carp. Significant differences (*p* < 0.05) among groups are indicated by different letters above the bars.

Consistent with the biochemical assays, the transcriptional abundances of *TGF-β1* and *IL-10* were remarkably upregulated by Zn supplementation relative to the unsupplemented control (*p* < 0.05). Specifically, *TGF-β1* transcription climbed to its peak within the Zn40–Zn160 range, maintaining a stable plateau without statistical variance among these higher-dose groups (*p* > 0.05). Specifically, *IL-10* transcription was significantly upregulated across the Zn20–Zn80 range relative to the Zn0 and Zn10 treatments (*p* < 0.05), forming a statistical plateau (*p* > 0.05) before experiencing a mild decline in the Zn160 cohort (*p* < 0.05). Conversely, the mRNA abundances of pro-inflammatory cytokines (*IL-6* and *IL-8*) were robustly repressed by Zn inclusion. Relative to the unsupplemented control, *IL-6* expression was universally downregulated across all treatments (*p* < 0.05), hitting its nadir in the Zn80 group (*p* < 0.05) before exhibiting a significant rebound at the maximum dosage (Zn160) (*p* < 0.05). Likewise, *IL-8* transcription was notably reduced in the Zn10–Zn160 groups relative to Zn0 (*p* < 0.05) and reached the minimum at Zn80 (*p* < 0.05), whereas the differences among Zn20, Zn40, Zn80 and Zn160 were generally not significant (*p* > 0.05). Meanwhile, Zn supplementation also downregulated *CAS-1* and *CAS-9* compared with Zn0 group (*p* < 0.05). The mRNA abundance of *CAS-1* hit its lowest point in the Zn40–Zn80 cohorts (*p* < 0.05) before rebounding at Zn160 (*p* < 0.05). A comparable trajectory was noted for *CAS-9*, which reached its minimum at Zn40 (*p* < 0.05). Although its expression climbed in the higher Zn groups (Zn80–Zn160), it remained markedly lower than the unsupplemented control (*p* < 0.05). Furthermore, *MAPK14* transcription level was markedly decreased in the Zn20 and Zn40 treatments in comparison with the Zn0 treatment (*p* < 0.05), followed by a partial restoration at the highest Zn doses (*p* < 0.05). Overall, dietary Zn enhanced anti-inflammatory signals while suppressing pro-inflammatory mediators, and 40 mg/kg Zn generally produced the most favorable transcriptional profile (*p* < 0.05)*,* Figure 6).

### 3.8. Histomorphometry and Intestinal Barrier-Related Genes’ Expression

**Figure 7:**
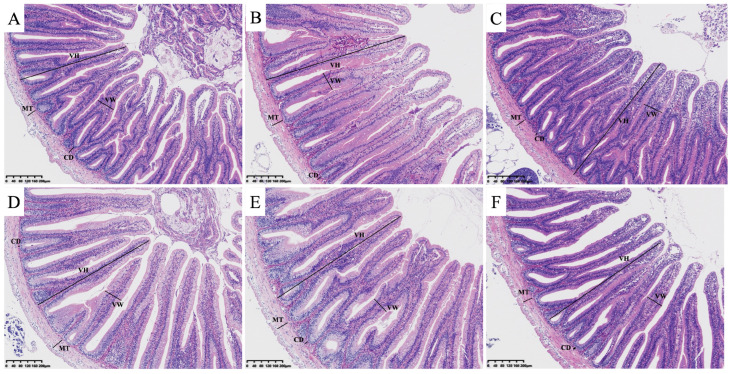
HE staining of the intestine sections of juvenile black carp fed with Zn0 (**A**), Zn10 (**B**), Zn20 (**C**), Zn40 (**D**), Zn80 (**E**), and Zn160 (**F**) (magnification ×80). VH: villus height, VW: villus width, MT: muscular thickness, CD: crypt depth.

Dietary Zn significantly influenced intestinal morphology (Table 10). Relative to the Zn0 group, villi height was significantly increased in all Zn treatments (*p* < 0.05). Villi width peaked in the Zn10 group (*p* < 0.05), while muscular thickness and crypt depth reached their maximums in the Zn80 group (*p* < 0.05) (Figure 7). However, excessive Zn supplementation (Zn160) caused significant reductions in villi width and crypt depth compared to the control (*p* < 0.05).

**Figure 8:**
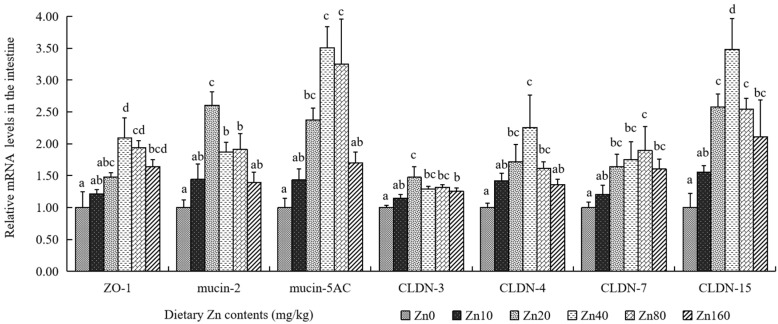
Effect of different levels of zinc on expression levels of barrier-related genes in the intestine of juvenile black carp. Significant differences (*p* < 0.05) among groups are indicated by different letters above the bars.

The transcription levels of *ZO-1*, *mucin-2*, *mucin-5AC*, *CLDN-3*, *CLDN-4*, *CLDN-7* and *CLDN-15* were notably heightened in the intestine of the Zn20, Zn40 and Zn80 treatment groups compared with the Zn0 treatment group (*p* < 0.05) (Figure 8). There were also no marked differences on the mRNA expression levels of *ZO-1*, *mucin-5AC*, *CLDN-4*, *CLDN-7* and *CLDN-9* among Zn20, Zn40 and Zn80 treatments (*p* > 0.05), while the transcription levels of *mucin-2*, *mucin-5AC*, *CLDN-4*, *CLDN-7*, *CLDN-9*, and *CLDN-15* were universally downregulated in the Zn160 group in comparison with the Zn40 treatment group (*p* < 0.05), and the levels of *mucin-2*, *mucin-5AC*, and *CLDN-4* in the highest Zn group were not statistically different from those of the Zn-deficient control (Zn0).

## 4. Discussion

Zinc functions as an indispensable micronutrient that underpins the somatic growth of diverse aquatic species [9,28]. In the current study, broken-line regression analysis of both WG and SGR revealed the optimal dietary Zn requirement for juvenile black carp (*M. piceus*) ranges from 44.59 to 44.63 mg/kg. Systematic comparison across fish species reveals interesting patterns in Zn requirements reflecting differences in phylogeny, feeding ecology, and metabolism [29]. The Zn requirement of black carp (*M. piceus*) was higher than that of yellow catfish (*Pelteobagrus fulvidraco*) [30] and rainbow trout (*Oncorhynchus mykiss*) [31], similar to grass carp (*Ctenopharyngodon idella*) [32] and Jian carp (*Cyprinus carpio* var. Jian) [33], but lower than that of largemouth bass (*Micropterus salmoides*) [34]. These variations might be attributed to multiple factors, including basal dietary Zn content, Zn source bioavailability, growth stage, metabolic rate, and species-specific digestive physiology [29,35,36]. The relatively high Zn requirement in black carp might be related to its specialized molluscivorous feeding habit, which demands enhanced synthesis of digestive enzymes and intestinal absorptive capacity to process hard-shelled prey.

Mechanistically, the growth-promoting properties of Zn are multifaceted, intricately linked to the optimized assimilation of nutrients and energy metabolism. Although no marked effects were presented on the contents of crude protein and ash in the whole fish body, fish body lipid content was significantly decreased at optimal and higher contents of dietary Zn, consistent with findings in yellow catfish (*P. fulvidraco* Richardson) [30]. The improved FCR suggests that adequate Zn levels in feed can enhance feed utilization efficiency and nutrient assimilation [30]. The liver and intestine are crucial organs for digestive enzyme secretion and nutrient absorption [37,38]. Zn plays key roles in the synthesis and activity regulation of digestive enzymes, which are closely related to digestive, metabolic, absorptive and growth processes in all fish species [39]. Previous research has found Zn-deficiency feeding could significantly reduce activities of TRY, CYT and AMS in pig [40], yellow catfish (*P. fulvidraco* Richardson) [30] and Siberian sturgeon (*Acipenser baerii*) [39], which is consistent with the present experimental results. Together with the improved FCR and overall growth, these findings suggest that adequate Zn inclusion significantly enhances digestive enzyme activities, thereby driving the superior growth performance of juvenile black carp.

Cellular Zn homeostasis is precisely maintained through coordinated regulation of Zn transporter (ZnT/*Slc30a*) families, which control Zn movement across cellular membranes in response to different levels of dietary Zn [41,42]. In teleosts, ZnT-regulating responses have been documented in zebrafish (*Danio rerio*) embryos and grass carp [43,44,45]. In the present study, optimal dietary Zn supplementation significantly upregulated the transcription of key zinc transporter genes (*ZNT1*, *ZNT2*, and *ZNT5*) in both the hepatic and intestinal tissues. This indicates that optimal dietary Zn supplementation is associated with the modulation of *ZnT* expression profiles to maintain integrated Zn homeostasis in black carp [37]. Conversely, relative lower expression levels of *ZNT1*, *ZNT2*, and *ZNT5* were observed in the Zn160 group, aligning with observations in yellow catfish [37]. This transcriptional downregulation under high-Zn conditions may serve as a protective mechanism to limit further Zn influx and avoid heavy metal overload in fish [46,47].

Serum biochemical indicators have been widely used to evaluate the metabolic homeostasis and nutritional status of fish [48]. Numerous studies have shown that relatively higher serum ALT, ALP, and AST activities can reflect the functional status of metabolic organs, particularly the liver [26,49,50]. Hepatic injury compromises hepatocyte membrane integrity, leading to the leakage of intracellular enzymes and a subsequent elevation in serum ALT and AST activities [51]. In this study, activities of ALT and AST declined in the serum of Zn-supplemented groups, which agrees with earlier findings in soft-shelled turtle (*Pelodiscus sinensis*) [52], indicating that optimal dietary Zn could improve the health status of hepatic cells in black carp (*M. piceus*) [53]. Functionally, HDL-C mediates reverse cholesterol transport from peripheral tissues to hepatic cells for subsequent catabolism, while LDL-C delivers hepatic cholesterol to other tissues for cellular utilization [54]. As we know, cholesterol can be coupled with bile acids to generate related mixtures and play important roles in lipid digestion and absorption [55]. Similar to results in soft-shelled turtle (*P. sinensis*) [52], higher HDL-C levels coupled with lower LDL-C levels were observed in these Zn-supplemented groups, indicating that adequate dietary Zn might be beneficial for serum lipid transport into hepatic cells in black carp (*M. piceus*). As the primary lipid molecules synthesized in the liver, TG and TC play indispensable roles in driving lipid metabolism and maintaining systemic metabolic homeostasis [56]. However, there was no significant difference in TG and TC levels in the Zn-added groups, which differs from results in soft-shelled turtle (*P. sinensis*) [52] and largemouth bass (*M. salmoides*) [57]. These divergences may be mediated by differences in fish species. Additionally, ALP is a Zn-dependent enzyme, and its significantly increased activity in the Zn-added groups indirectly reflects improved Zn metabolism and utilization [58], consistent with findings in golden pompano (*Trachinotus ovatus*) [59]. Furthermore, serum GLU levels were notably decreased in the Zn-added groups, consistent with previous findings in *Pangasius hypophthalmus* [60] and largemouth bass (*M. salmoides*) [57]. This hypoglycemic effect likely stems from Zn-mediated regulation of glucose metabolism, though further metabolic studies are required to fully elucidate this mechanism in fish.

It is widely recognized that reactive oxygen species (ROS) are continuously generated during the aerobic metabolism of nutrients, including Zn, which significantly influences redox homeostasis in animals [61,62]. Acting as crucial components of the antioxidant defense system, key enzymes such as SOD, CAT, and GPX catalyze the conversion of toxic oxygen radicals into harmless H_2_O and O_2_, thereby mitigating oxidative stress in fish [55,63]. In juvenile black carp (*M. piceus*), the activities of T-SOD, GPX, and CAT were notably upregulated in both hepatic and intestinal tissues following dietary Zn supplementation. Similar Zn-induced enhancements in these antioxidant enzymes have been widely reported in other teleosts, including rainbow trout (*Oncorhynchus mykiss*) [64], Jian carp (*Cyprinus carpio* var. Jian) [65], half-smooth tongue sole (*Cynoglossus semilaevis*) [66], and golden pompano (*T. ovatus*) [59]. These consistent results strongly suggest that adequate dietary Zn plays a vital role in activating and bolstering the primary enzymatic antioxidant defense system in fish [64]. Beyond primary enzymes, the glutathione system is essential for sustaining cellular redox balance. GR facilitates the reduction of glutathione disulfide (GSSG) into GSH [67], while GST drives the generation of glutathione S-conjugates for detoxification [68]. Elevated GST typically serves as an adaptive physiological response to oxidative damage or inflammatory stimuli [69]. In our study, Zn supplementation significantly increased GSH content and T-AOC in both the liver and intestine. The observed decrease in GST alongside the increase in GR activity suggests that Zn promotes the regeneration of reduced GSH, thereby expanding the cellular antioxidant pool to maintain redox homeostasis [65]. Under oxidative stress, excessive ROS damages cell membranes, leading to lipid peroxidation, which is reliably quantified by MDA content [70]. As a vital antioxidant, GSH scavenges oxygen radicals and organic peroxides, protecting tissues and preventing harmful MDA accumulation [71]. Previous studies confirm that dietary Zn levels significantly affect GSH synthesis and effectively reduce MDA concentrations [72,73]. Consistently, our results showed that Zn supplementation significantly reduced hepatic and intestinal MDA levels, aligning with findings in Jian carp (*C. carpio var. Jian*) [65] and golden pompano (*T. ovatus*) [59]. At the molecular level, these biochemical adaptations are governed by the *Nrf2/Keap1* axis, a master regulator of cellular antioxidant responses [74,75]. Dietary Zn supplementation not only significantly enhanced *Nrf2* transcription but also downregulated *Keap1* mRNA expression, corroborating previous results in yellow catfish (*Pelteobagrus fulvidraco*) [73]. Taken together, our findings demonstrate that adequate dietary Zn enhances antioxidant capacity and mitigates lipid peroxidation in black carp by stimulating antioxidant enzyme activities and increasing cellular GSH content, a process likely mediated through the activation of the Nrf2/Keap1 signaling pathway.

The integrity of the intestinal physical barrier is primarily governed by the tight junction (TJ) complex, including zonula occludens (ZOs) and claudins (CLDNs), alongside the chemical barrier formed by mucins [25,76]. These proteins act as essential biomarkers for evaluating the functional integrity of the intestinal barrier [77,78], and are indispensable for regulating epithelial permeability and supporting external nutrient absorption [79]. Specifically, as a peripheral membrane protein, ZO-1 is fundamental in constructing and stabilizing tight junctions between epithelial cells [80]. Concurrently, CLDNs function as key transmembrane components that sustain cell-to-cell barriers, manage intercellular communication, and preserve cellular polarity [81]. In our present study, optimal dietary Zn supplementation, particularly in the Zn40 group, markedly upregulated the mRNA expressions of *ZO-1*, as well as multiple claudin members (*CLDN-3*, *CLDN-4*, *CLDN-7*, and *CLDN-15*). Furthermore, the transcriptions of *mucin-2* and *mucin-5AC*, which act as crucial components of the intestinal chemical barrier, exhibited a synchronized upregulation under adequate Zn intake. This observation is highly consistent with prior investigations in Nile tilapia (*Oreochromis niloticus*) [82], rainbow trout (*O. mykiss*) [83], and grass carp (*C. idella*) [45]. These transcriptional profiles indicate that an adequate dietary intake of Zn is associated with improved intestinal barrier function by simultaneously reinforcing the physical barrier (*ZO-1* and *CLDNs*) and supporting chemical mucus secretion.

A growing body of evidence indicates that augmenting the levels of key immune effectors fundamentally bolsters host immunity, including complement components (C3, C4, and CFB) and humoral immune-related genes (*LZM*, *NRAMP*, *IgH*, and *Igλ1*) [57,77,84] In the present study, Zn-supplemented diets effectively counteracted potential damage by enhancing intestinal immunity, evidenced by increased C3, C4, IgM, and LZM activities. This immunomodulatory effect of adequate Zn aligns strongly with similar findings reported in largemouth bass (*M. salmoides*) [57]. Furthermore, appropriate dietary Zn content significantly increased intestinal ACP and ALP activities, aligning with findings in rats [85] and various fish species, such as largemouth bass (*M. salmoides*) juveniles supplemented with dietary soybean lecithin [77] and black carp (*M. piceus*) fed selenium yeast [26]. It is extensively indicated in the literature that Zn deprivation triggers metabolic stress and physiological disturbances [86], ultimately compromising the survival, proliferation, and maturation of immune cells [87]. Central to regulating these immune responses and inflammatory processes is the strict homeostatic balance of inflammatory cytokines [86]. In our results, *IL-10* and *TGF-β1*, two typical anti-inflammatory cytokines, showed increasing then stabilizing trends with dietary Zn increases, similar to results in largemouth bass (*M. salmoides*) treated with dietary Zn [74]. Conversely, the expression levels of pro-inflammatory cytokines (*IL-1β*, *IL-6*, *IFN-γ*, and *TNF-α*) exhibited a pattern of initial suppression followed by a plateau, corroborating previous observations in largemouth bass (*M. salmoides*) [74], indicating adequate dietary Zn could suppress the contents of these pro-inflammatory cytokines at both transcriptional and protein levels in black carp (*M. piceus*).

Crucially, inflammatory and apoptotic responses are meticulously regulated by distinct signaling cascades. In our study, adequate dietary Zn downregulated the mRNA expression of *CAS-1* and *CAS-9*. It is well established that Caspase-1 plays a vital role in the inflammasome complex, facilitating the maturation and secretion of IL-1β, whereas Caspase-9 functions primarily as an initiator caspase in the mitochondrial apoptosis pathway [74]. The concomitant reduction in *CAS-1* and *IL-1β* transcription suggests that adequate Zn may help alleviate intestinal inflammation, while the downregulation of *CAS-9* points to a potential attenuation of cellular apoptosis. Mechanistically, the production of pro-inflammatory cytokines is largely governed by the p38 MAPK signaling cascade. Our data showed that adequate Zn supplementation downregulated *MAPK14* mRNA expression, similar to findings in largemouth bass [74]. Although further investigation at the protein phosphorylation level is required to confirm functional pathway activity, the current transcriptional evidence suggests that adequate dietary Zn may be involved in mitigating intestinal inflammation, potentially by modulating the p38 MAPK cascade and Caspase-1 signaling in black carp.

## 5. Conclusions

This study demonstrates that the optimal dietary zinc requirement for juvenile black carp (*M. piceus*) is approximately 44.6 mg/kg, based on growth performance. Adequate dietary Zn supplementation significantly improves feed utilization, reduces whole-body lipid deposition, and enhances digestive enzyme activities. Furthermore, optimal Zn intake is associated with the modulation of Zn transporter gene expression to maintain trace element homeostasis. It also upregulates antioxidant enzyme activities and related gene expressions potentially involving the Nrf2/Keap1 pathway, bolsters intestinal barrier integrity by promoting tight junction and mucin transcription, and enhances innate immunity. Importantly, adequate dietary Zn could exert an anti-inflammatory effect, which may be associated with the transcriptional modulation of the *MAPK14* and *CAS-1* signaling cascades. From a practical perspective, maintaining a suitable Zn inclusion level in commercial aquafeeds is highly recommended to maximize feed efficiency, ensure robust intestinal health, and improve the economic sustainability of black carp culture.

## Figures and Tables

**Table 1:** Ingredient and proximate composition of the experimental diets (on dry weight basis).

Ingredients	Dietary Zinc Levels (g/kg)					
	Zn0	Zn10	Zn20	Zn40	Zn80	Zn160
Casein ^a^	380.00	380.00	380.00	380.00	380.00	380.00
Gelatin ^b^	60.00	60.00	60.00	60.00	60.00	60.00
Dextrin ^b^	250.00	250.00	250.00	250.00	250.00	250.00
Fish oil ^c^	54.00	54.00	54.00	54.00	54.00	54.00
Microcrystalline cellulose ^b^	150.50	150.46	150.41	150.32	150.14	149.79
Zn-free mineral premix ^d^	25.00	25.00	25.00	25.00	25.00	25.00
Vitamin premix ^e^	12.50	12.50	12.50	12.50	12.50	12.50
Attractant ^f^	20.00	20.00	20.00	20.00	20.00	20.00
Choline chloride ^c^	3.00	3.00	3.00	3.00	3.00	3.00
ZnSO_4_·7H_2_O ^g^	0.00	0.04	0.09	0.18	0.36	0.71
Proximate composition						
Crude protein	381.26	389.73	390.08	388.69	386.37	392.05
Crude lipid	63.65	60.00	58.21	59.49	58.08	63.06
Ash	24.30	24.00	24.30	24.00	24.10	24.30

Note: ^a^ Casein, obtained from Gansu Hualing Dairy Co., Ltd., Hezuo, China, crude protein 80.56%. ^b^ Gelatin, Potato starch and Microcrystalline cellulose, obtained from Sinopharm Chemical Reagent Co., Ltd., Shanghai, China, crude protein 87.17%. ^c^ Zhejiang Yixing Feed Group Co. Ltd., Hangzhou, China. ^d^ Zn-free mineral mixtures (g/Kg diet): NaSeO_3_, 1.2 mg; KI, 2 mg; CoCl_2_·6H_2_O, 13 mg; CuSO_4_·5H_2_O, 5 mg; FeSO_4_·7H_2_O, 70 mg; MnSO_4_·H_2_O, 45 mg; MgSO_4_·7H_2_O, 130 mg; NaCl, 50 mg. ^e^ Vitamin premix (g/Kg diet): vitamin A, 20 mg; vitamin D_3_, 3 mg; vitamin C, 1000 mg; vitamin E, 300 mg; thiamin, 30 mg; riboflavin, 10 mg; pyridoxine, 20 mg; vitamin B_12_, 0.2 mg; vitamin K_3_, 5 mg; inositol, 1000 mg; pantothenic acid, 30 mg; folic acid, 3 mg; niacin acid, 50 mg; biotin, 1 mg. ^f^ Attractant composition: taurine:betain-HCl:glycine = 1:3:3. ^g^ Zinc sulfate heptahydrate, obtained from Chengdu Shuxing Feed Co., Ltd. (Chengdu, China). Purity: 99.7%; Zn content: 34.5%.

**Table 2:** Primer sequences for real-time PCR analysis.

Gene	Forward Primer (5′-3′)	Reverse Primer (5′-3′)	Product Sizes (bp)
*Cu/Zn-SOD*	GCAGGTCCGCACTTCAA	GACAGGGACAGCATTTGGT	143
*Mn-SOD*	GCAGGGCACTACAGGTCTC	CTCCCAGTTCACAACATTCC	124
*CAT*	CAGTATCTTACGTGATGGGTCT	GGAAGTTGCCGTTGGAGAT	101
*GPX1*	GCAACCAGTTCGGACATCA	GCGTTCTCACCGTTCACTT	133
*GR*	CAGAACACTACACGTCCAGG	CAAACAGTCGGTGAGCAAG	109
*Nrf2*	TGTCCAAACACCAGCTCAA	CGTACTCTAGGCCCACGAT	129
*Keap1*	GGCTGCTCTGTGATCTGGTT	TTCCTTGAAGTTGCTGGTGA	128
*C3*	CAAGTGGCTGGTTCTCAA	ATGGCAATCACAATAAAGG	138
*C4*	GGAGGTATTGGAGGAGTTGA	GAAGTTGGTGGCACGAGA	132
*CFB*	GAATCCCATACCTAAAGTGAACC	GTCCCTCTGTGACACCAAGCT	142
*LZM*	TGCCTTGTTCAGATTTGCT	GTAACTATCCCAGGTGTCCC	107
*IgH*	CTGGTCTGTACCGCCTCTG	CTGGACTGACTGGGAATAGG	138
*Ig* *λ* *1*	TGTGACAGACCAAGGGAAAG	TGACTGGCGTGGCATAAC	105
*IL-6*	GCCAGCTCCAGGTGAGTGA	CAGGATCGAGTGTACGGTTG	101
*IL-8*	TGCTGCCGTCTGCTGCTT	CGAGGTGGGATTACGGATGA	108
*TGF-β1*	AAATGGGTGGCAAGTGGGT	TGTTCTTCTAAGATGGCTTTATCG	112
*IL-10*	CCTTTGAGTTTGCCACCC	TTTGATGCCAGATACTGTTCG	118
*CAS-1*	CAATGTCAGGGTCCGAAGG	TTGTTGGCTGCATGGAGTAA	102
*CAS-9*	CATCCTGGTGTCCTACTCAACC	GTGGCAACATTCTCCTCAAGC	111
*MAPK14*	TGAGGGTCGCAGTCAAGAAG	ACTGGTAGCGGGCGAGAAA	143
*ZNT1*	GGCAGGCAGTCGCATTATCG	TGGATGCCCTCATCATGGAAA	108
*ZNT2*	AGAAGTCATTGGTGGATACGCT	TTGGAAGGTGGTCTGGAAGA	129
*ZNT5*	AATGAATGCTAATATGCGAGGAG	TGTGGCAATGAAGAGTGAACAA	142
*ZNT9*	GGCGTCAAATACACCCAGAAT	ATCGCAGGAAGACGGTAAAGG	148
*Trx*	CGCTCTTCGTCATTCTCATCCTG	GGTGAAGTCGTCGTGGTCCTG	126
*TrxR*	ACACCAAAGGGCACAACCTG	AACTCCCAGCCGAACTTGC	131
*ZO-1*	GATGCCGAAAGAGCAGAGTGC	ACAGGCAGGTCCACGTCAGG	108
*Mucin-2*	TGTGATGACTCCTCTTTGTTGGCTCTA	CGGGCTGTTGAAATGATTGTCG	144
*Mucin-5AC*	ATGCCTAAATGAAATGAATGGACC	GCTGCTAAACTGCCGAAGAG	132
*CLDN-3*	GCACAGGTGTACTGGGAGGGA	CCGCAGGAAGAGCCAACATA	100
*CLDN-4*	TTCCTCATCCTCGTACCCGTCTG	AATCAACAGCAATGCCGAACCTC	141
*CLDN-7*	GCCAGCATGGGCATGAAGT	GTAAACCAGCCACAGGCAACA	131
*CLDN-15*	GGCTGGAGGCGTCTTCTTC	GCCCTTCTCCGATTTCATACTT	131
*β-actin*	CCTTCTTGGGTATGGAGTC	GTCAGCAATGCCAGGGTA	140

Note: *Cu/Zn-SOD*, Cu/Zn-Superoxide dismutase; *Mn-SOD*, Mn-Superoxide dismutase; *CAT*, catalase; *GPX1*, glutathione peroxidase 1; *GR*, glutathione reductase; *Trx*, thioredoxin; *TrxR*, thioredoxin reductase; *Nrf2*, NF-E2-related factor 2; *Keap1*, kelch-like ECH-associated protein 1; *C3*, complement component 3; *C4*, complement component 4; *CFB*, complement factor B; *LZM*, lysozyme; *IgH*, immunoglobulin mu heavy chain; *Igλ1*, immunoglobulin lambda-1; *IL-6*, interleukin 6; *IL-8*, interleukin 8; *TGF-β1*, transforming growth factor 1beta; *CAS-1*, caspase-1; *CAS-9*, caspase-9; mitogen-activated protein kinase 14; *ZNT1*, zinc transporter 1; *ZNT2*, zinc transporter 2; *ZNT5*, zinc transporter 5; *ZNT9*, zinc transporter 9; *ZO-1*, zonula occludens-1; *CLDN-3*, claudin 3; *CLDN-4*, claudin 4; CLDN-7, claudin 7; *CLDN-15*, claudin 15.

**Table 3:** Effects of different levels of zinc on growth performance of juvenile black carp (mean ± SD). Means in each row with different superscripts show a significant difference (*p* < 0.05).

Items	Dietary Zinc Levels (g/kg)					
	Zn0	Zn10	Zn20	Zn40	Zn80	Zn160
Growth and feed performance						
IBW (g)	2.94 ± 0.06	2.79 ± 0.06	2.83 ± 0.03	2.84 ± 0.11	2.90 ± 0.16	2.87 ± 0.19
FBW(g)	15.09 ± 0.75 ^a^	16.22 ± 0.23 ^b^	20.03 ± 1.28 ^c^	19.74 ± 0.51 ^c^	19.84 ± 1.00 ^c^	19.25 ± 1.02 ^c^
WG (%)	416.08 ± 13.39 ^a^	492.53 ± 13.73 ^b^	601.43 ± 12.90 ^c^	595.46 ± 12.97 ^c^	600.65 ± 13.51 ^c^	595.31 ± 14.75 ^c^
SGR (%/d)	2.62 ± 0.04 ^a^	2.82 ± 0.04 ^b^	3.18 ± 0.07 ^c^	3.11 ± 0.06 ^c^	3.05 ± 0.08 ^c^	3.06 ± 0.09 ^c^
FCR	1.94 ± 0.06 ^b^	1.74 ± 0.07 ^a^	1.69 ± 0.05 ^a^	1.65 ± 0.04 ^a^	1.72 ± 0.07 ^a^	1.67 ± 0.05 ^a^
HSI (%)	1.87 ± 0.09	1.97 ± 0.26	1.86 ± 0.07	1.74 ± 0.16	1.87 ± 0.06	1.87 ± 0.08
CF (%)	1.74 ± 0.04	1.75 ± 0.05	1.82 ± 0.06	1.76 ± 0.04	1.75 ± 0.03	1.76 ± 0.06
Body composition						
Crude protein (%)	15.57 ± 0.58	15.81 ± 0.40	15.28 ± 0.51	15.5 ± 0.20	15.74 ± 0.40	15.66 ± 0.42
Crude lipid (%)	8.68 ± 0.20 ^c^	8.06 ± 0.15 ^b^	7.62 ± 0.10 ^a^	7.73 ± 0.11 ^a^	7.67 ± 0.23 ^a^	7.66 ± 0.13 ^a^
Ash (%)	2.76 ± 0.05	2.74 ± 0.05	2.70 ± 0.07	2.71 ± 0.07	2.77 ± 0.07	2.71 ± 0.04
Zn (mg/kg)	53.05 ± 0.25 ^a^	76.57 ± 0.76 ^b^	84.02 ± 1.56 ^c^	86.60 ± 1.65 ^cd^	87.58 ± 1.11 ^d^	87.57 ± 1.19 ^d^

Note: IBW (g), initial body weight; FBW (g), final body weight; Weight gain (WG, %) = (final body weight − initial body weight)/initial body weight × 100; Specific growth rate (SGR, %/d) = (ln final body weight − ln initial body weight) × 100/days; Feed conversion ratio (FCR) = dry feed consumed/(final body weight − initial body weight); Hepatosomatic index (HSI, %) = liver weight/final body weight × 100; Condition factor (CF, %) = final body weight (g)/length (cm)^3^ × 100. The lowercase letters (a, b, c, d) denote significant differences among treatments (*p* < 0.05).

**Table 4:** Effects of dietary zinc on the serum biochemical indicators in juvenile black carp (mean ± SD, *n* = 3). Means in each row with different superscripts show significant difference (*p* < 0.05).

Items	Dietary Zinc Levels (mg/kg)					
	Zn0	Zn10	Zn20	Zn40	Zn80	Zn160
HDL-C (mmol/L)	0.41 ± 0.03 ^a^	0.48 ± 0.04 ^ab^	0.49 ± 0.05 ^b^	0.49 ± 0.04 ^b^	0.49 ± 0.03 ^b^	0.50 ± 0.04 ^b^
LDL-C (mmol/L)	1.67 ± 0.12 ^b^	1.51 ± 0.05 ^a^	1.51 ± 0.06 ^a^	1.51 ± 0.04 ^a^	1.51 ± 0.04 ^a^	1.52 ± 0.02 ^a^
TG (mmol/L)	4.29 ± 0.19	4.25 ± 0.30	4.31 ± 0.21	4.33 ± 0.26	4.29 ± 0.12	4.34 ± 0.07
TC (mmol/L)	3.74 ± 0.18	3.81 ± 0.30	3.80 ± 0.15	3.76 ± 0.29	3.73 ± 0.09	3.76 ± 0.10
GLU (mmol/L)	5.31 ± 0.26 ^c^	4.80 ± 0.22 ^b^	3.59 ± 0.15 ^a^	3.51 ± 0.17 ^a^	3.51 ± 0.26 ^a^	3.64 ± 0.21 ^a^
AST (U/L)	509.30 ± 6.66 ^b^	426.10 ± 8.65 ^a^	425.53 ± 9.11 ^a^	427.90 ± 7.65 ^a^	427.83 ± 7.46 ^a^	421.27 ± 9.39 ^a^
ALT (U/L)	25.87 ± 2.35 ^c^	17.97 ± 1.75 ^ab^	17.47 ± 1.23 ^a^	18.00 ± 1.51 ^ab^	18.07 ± 1.21 ^ab^	21.17 ± 1.79 ^b^
ALP (U/L)	261.60 ± 16.95 ^a^	328.33 ± 18.95 ^b^	326.97 ± 13.46 ^b^	327.13 ± 17.83 ^b^	318.67 ± 11.14 ^b^	320.9 ± 11.23 ^b^
ALB (g/L)	10.40 ± 0.80	11.00 ± 0.62	9.87 ± 0.55	10.37 ± 0.85	9.90 ± 0.78	10.2 ± 0.89
TBA (μmol/L)	4.93 ± 0.15	4.97 ± 0.21	5.03 ± 0.21	5.00 ± 0.20	5.03 ± 0.15	4.93 ± 0.15

Note: The lowercase letters (a, b, c) denote significant differences among treatments (*p* < 0.05).

**Table 5:** Effect of different levels of zinc on the activities of metabolic enzymes in the liver and intestine of juvenile black carp. Means in each row with different superscripts show a significant difference (*p* < 0.05).

Items	Dietary Zinc Levels (mg/kg)					
	Zn0	Zn10	Zn20	Zn40	Zn80	Zn160
Liver						
LPS (U/g prot)	2.02 ± 0.05 ^a^	2.14 ± 0.02 ^ab^	2.17 ± 0.12 ^b^	2.16 ± 0.06 ^b^	2.17 ± 0.09 ^b^	2.17 ± 0.04 ^b^
AMS (U/g prot)	12.07 ± 0.15 ^a^	14.11 ± 0.22 ^b^	17.10 ± 0.55 ^c^	17.36 ± 0.32 ^c^	17.08 ± 0.40 ^c^	17.79 ± 0.87 ^c^
TRY (U/g prot)	268.39 ± 54.69 ^a^	440.13 ± 30.49 ^b^	495.60 ± 30.66 ^b^	498.28 ± 81.55 ^b^	482.05 ± 26.09 ^b^	488.58 ± 61.07 ^b^
CYT (U/mg prot)	4.58 ± 0.51 ^a^	7.04 ± 1.43 ^ab^	7.47 ± 2.03 ^b^	8.35 ± 1.69 ^b^	9.81 ± 1.43 ^b^	9.23 ± 1.22 ^b^
Intestine						
LPS (U/g prot)	9.49 ± 0.14 ^a^	11.04 ± 0.32 ^b^	11.11 ± 0.11 ^b^	11.15 ± 0.18 ^b^	10.96 ± 0.49 ^b^	11.25 ± 0.38 ^b^
AMS (U/g prot)	42.92 ± 0.64 ^a^	52.25 ± 0.81 ^b^	52.63 ± 0.23 ^b^	52.85 ± 0.86 ^b^	52.46 ± 0.75 ^b^	52.46 ± 0.76 ^b^
TRY (U/g prot)	75.15 ± 13.42 ^a^	110.64 ± 14.56 ^a^	192.55 ± 22.17 ^b^	210.64 ± 14.43 ^b^	208.92 ± 32.78 ^b^	190.09 ± 24.33 ^b^
CYT (U/mg prot)	15.33 ± 1.63 ^a^	25.43 ± 2.06 ^b^	30.61 ± 2.76 ^b^	33.48 ± 1.77 ^c^	32.51 ± 4.76 ^c^	32.20 ± 4.31 ^c^

Note: The lowercase letters (a, b, c) denote significant differences among treatments (*p* < 0.05).

**Table 6:** Effect of different levels of zinc on the activities of antioxidant enzymes in the liver of juvenile black carp (mean ± SD). Means in each row with different superscripts show a significant difference (*p* < 0.05).

Items	Dietary Zinc Levels (mg/kg)					
	Zn0	Zn10	Zn20	Zn40	Zn80	Zn160
T-SOD (U/mg prot)	98.11 ± 1.57 ^a^	109.34 ± 2.58 ^b^	113.79 ± 1.34 ^c^	114.11 ± 1.76 ^c^	115.62 ± 2.97 ^c^	115.6 ± 2.45 ^c^
CAT (U/mg prot)	56.93 ± 3.07 ^a^	83.29 ± 2.66 ^b^	143.16 ± 4.18 ^c^	142.71 ± 5.80 ^c^	137.66 ± 6.99 ^c^	146.83 ± 5.96 ^c^
GPX (U/mg prot)	5.36 ± 0.69 ^a^	12.68 ± 0.53 ^b^	17.54 ± 1.06 ^d^	13.32 ± 0.90 ^bc^	14.54 ± 1.13 ^c^	13.43 ± 0.60 ^bc^
GR (U/g prot)	1.63 ± 0.06 ^a^	1.77 ± 0.03 ^ab^	1.83 ± 0.07 ^bc^	1.84 ± 0.07 ^bc^	1.91 ± 0.18 ^bc^	1.97 ± 0.11 ^c^
GST (U/mg prot)	19.63 ± 0.70 ^c^	16.43 ± 0.51 ^ab^	15.9 ± 0.64 ^a^	16.88 ± 0.16 ^b^	16.77 ± 0.25 ^ab^	16.22 ± 0.43 ^ab^
GSH (μmol/g prot)	235.99 ± 3.65 ^a^	263.91 ± 1.44 ^b^	271.19 ± 3.15 ^c^	273.71 ± 4.37 ^c^	270.29 ± 3.65 ^c^	272.01 ± 3.38 ^c^
T-AOC (mmol/g prot)	0.38 ± 0.01 ^a^	0.43 ± 0.01 ^b^	0.45 ± 0.01 ^b^	0.45 ± 0.01 ^b^	0.45 ± 0.01 ^b^	0.44 ± 0.03 ^b^
MDA (nmol/mg prot)	0.99 ± 0.11 ^c^	0.80 ± 0.04 ^b^	0.61 ± 0.07 ^a^	0.58 ± 0.05 ^a^	0.60 ± 0.05 ^a^	0.60 ± 0.08 ^a^

Note: The lowercase letters (a, b, c, d) denote significant differences among treatments (*p* < 0.05).

**Table 7:** Effect of different levels of zinc on the activities of antioxidant enzymes in the intestine of juvenile black carp (mean ± SD). Means in each row with different superscripts show a significant difference (*p* < 0.05).

Items	Dietary Zinc Levels (mg/kg)					
	Zn0	Zn10	Zn20	Zn40	Zn80	Zn160
T-SOD (U/mg prot)	7.25 ± 0.66 ^a^	9.13 ± 0.83 ^b^	13.05 ± 0.45 ^c^	12.74 ± 0.42 ^c^	13.09 ± 0.52 ^c^	12.89 ± 0.37 ^c^
CAT (U/mg prot)	13.63 ± 1.61 ^a^	41.25 ± 2.48 ^b^	53.9 ± 3.78 ^c^	53.27 ± 1.93 ^c^	57.21 ± 5.81 ^c^	58.59 ± 3.82 ^c^
GPX (U/mg prot)	9.55 ± 1.13 ^a^	18.16 ± 1.21 ^b^	35.4 ± 1.57 ^c^	33.68 ± 2.64 ^c^	36.64 ± 3.60 ^c^	38.04 ± 3.51 ^c^
GR (U/g prot)	8.41 ± 0.50 ^a^	9.23 ± 0.67 ^a^	13.42 ± 0.85 ^b^	13.06 ± 0.33 ^b^	13.11 ± 0.53 ^b^	13.21 ± 0.87 ^b^
GST (U/mg prot)	40.17 ± 0.97 ^c^	23.44 ± 0.74 ^ab^	24.29 ± 0.32 ^b^	23.08 ± 0.23 ^a^	24.06 ± 0.73 ^ab^	24.32 ± 0.17 ^b^
GSH (μmol/g prot)	217.24 ± 5.04 ^a^	279.1 ± 5.07 ^b^	308.96 ± 8.60 ^c^	286.06 ± 4.12 ^b^	286.48 ± 5.19 ^b^	281.99 ± 5.37 ^b^
T-AOC (mmol/g prot)	0.36 ± 0.02 ^a^	0.50 ± 0.03 ^b^	0.50 ± 0.07 ^b^	0.47 ± 0.02 ^b^	0.51 ± 0.02 ^b^	0.54 ± 0.09 ^b^
MDA (nmol/mg prot)	15.61 ± 0.23 ^c^	10.52 ± 0.11 ^b^	6.73 ± 0.09 ^a^	6.87 ± 0.39 ^a^	7.04 ± 0.40 ^a^	6.79 ± 0.30 ^a^

Note: The lowercase letters (a, b, c) denote significant differences among treatments (*p* < 0.05).

**Table 8:** Effect of different levels of zinc on the activities of immune-related factors in the intestine of juvenile black carp (mean ± SD). Means in each row with different superscripts show a significant difference (*p* < 0.05).

Items	Dietary Zinc Levels (mg/kg)					
	Zn0	Zn10	Zn20	Zn40	Zn80	Zn160
LZM (U/mg prot)	283.1 ± 2.63 ^a^	291.69 ± 7.16 ^b^	334.1 ± 8.65 ^c^	324.01 ± 4.88 ^bc^	320.34 ± 7.16 ^b^	312.32 ± 8.65 ^b^
IgM (μg/mg prot)	2.08 ± 0.17 ^a^	4.27 ± 0.21 ^b^	4.66 ± 0.33 ^b^	4.64 ± 0.24 ^b^	4.54 ± 0.25 ^b^	4.33 ± 0.20 ^b^
ACP (U/g prot)	1.63 ± 0.04 ^a^	2.02 ± 0.02 ^b^	2.53 ± 0.06 ^c^	2.42 ± 0.06 ^c^	2.47 ± 0.07 ^c^	2.43 ± 0.12 ^c^
ALP (U/g prot)	5.34 ± 0.06 ^a^	5.66 ± 0.05 ^b^	6.26 ± 0.04 ^c^	6.26 ± 0.03 ^c^	6.24 ± 0.05 ^c^	6.24 ± 0.05 ^c^
C3 (μg/mg prot)	136.44 ± 14.70 ^a^	362.69 ± 14.42 ^b^	440.4 ± 10.94 ^c^	442.9 ± 14.34 ^c^	429.21 ± 11.98 ^c^	450.40 ± 24.61 ^c^
C4 (μg/mg prot)	36.80 ± 3.39 ^a^	84.80 ± 1.20 ^b^	95.8 ± 5.05 ^c^	97.58 ± 3.69 ^c^	97.91 ± 4.84 ^c^	94.80 ± 3.29 ^c^

Note: The lowercase letters (a, b, c) denote significant differences among treatments (*p* < 0.05).

**Table 9:** Effect of different levels of zinc on the activities of inflammation in the intestine of juvenile black carp (mean ± SD). Means in each row with different superscripts show a significant difference (*p* < 0.05).

Items	Dietary Zinc Levels (mg/kg)					
	Zn0	Zn10	Zn20	Zn40	Zn80	Zn160
IL-10 (ng/L)	123.40 ± 7.64 ^a^	138.62 ± 6.99 ^a^	232.10 ± 19.73 ^c^	227.75 ± 12.74 ^c^	182.82 ± 14.25 ^b^	190.80 ± 16.89 ^b^
TGF-β1 (pg/mL)	117.30 ± 9.42 ^a^	137.79 ± 2.49 ^b^	142.30 ± 7.98 ^b^	144.04 ± 5.29 ^b^	135.01 ± 6.75 ^b^	142.47 ± 4.30 ^b^
TNF-α (ng/L)	135.54 ± 5.01 ^c^	114.71 ± 8.09 ^b^	87.50 ± 5.75 ^a^	95.83 ± 6.12 ^a^	93.14 ± 7.79 ^a^	88.24 ± 7.46 ^a^
IL-1β (ng/L)	137.15 ± 14.23 ^c^	106.51 ± 12.46 ^b^	78.82 ± 16.95 ^a^	91.9 ± 9.12 ^ab^	89.59 ± 8.65 ^ab^	107.79 ± 9.74 ^b^
IL-6 (ng/L)	115.66 ± 17.75 ^c^	61.15 ± 13.01 ^b^	59.06 ± 4.78 ^b^	28.16 ± 1.59 ^a^	34.75 ± 6.28 ^a^	31.29 ± 7.02 ^a^
IFN-γ (ng/L)	64.69 ± 5.34 ^b^	45.42 ± 8.83 ^a^	43.83 ± 7.06 ^a^	43.03 ± 5.74 ^a^	46.29 ± 4.09 ^a^	36.00 ± 5.92 ^a^

Note: The lowercase letters (a, b, c) denote significant differences among treatments (*p* < 0.05).

**Table 10:** Effects of dietary zinc levels on intestinal morphology of juvenile black carp (mean ± SD). Means in each row with different superscripts show a significant difference (*p* < 0.05).

Items	Dietary Zinc Levels (mg/kg)					
	Zn0	Zn10	Zn20	Zn40	Zn80	Zn160
Villi height (μm)	634.0 ± 33.1 ^c^	872.1 ± 60.3 ^a^	748.0 ± 33.6 ^b^	752.2 ± 9.0 ^b^	848.3 ± 47.2 ^a^	801.1 ± 41.4 ^ab^
Villi width (μm)	80.6 ± 4.9 ^b^	110.5 ± 9.8 ^a^	89.3 ± 11.1 ^b^	88.7 ± 10.7 ^b^	93.3 ± 7.6 ^ab^	60.0 ± 14.8 ^c^
Muscular thickness (μm)	53.0 ± 5.0 ^b^	60.9 ± 8.3 ^ab^	62.8 ± 1.6 ^ab^	58.6 ± 4.9 ^ab^	68.9 ± 3.3 ^a^	59.6 ± 14.4 ^ab^
Crypt depth (μm)	12.4 ± 2.2 ^bc^	17.7 ± 2.9 ^ab^	14.0 ± 4.1 ^bc^	15.5 ± 4.3 ^abc^	21.7 ± 4.5 ^a^	9.7 ± 0.8 ^c^

## Data Availability

Data are contained within this article.

## Abbreviations

The following abbreviations are used in this manuscript:

Abbreviation	Full Name/Definition
ACP	Acid phosphatase
ALB	Albumin
ALP	Alkaline phosphatase
ALT	Alanine aminotransferase
AMS	Amylase
ANOVA	Analysis of variance
AST	Aspartate aminotransferase
C3/C4	Complement component 3/Complement component 4
CAS-1/CAS-9	Caspase-1/Caspase-9
CAT	Catalase
CD	Crypt depth
CF	Condition factor
CFB	Complement factor B
CLDN	Claudin (e.g., CLDN-3, CLDN-4)
Cu/Zn-SOD	Copper/zinc superoxide dismutase
CYT	Chymotrypsin
FBW	Final body weight
FCR	Feed conversion ratio
GLU	Glucose
GPX/GSH-PX	Glutathione peroxidase
GR	Glutathione reductase
GSH	Glutathione (reduced)
GSSG	Glutathione disulfide
GST	Glutathione S-transferase
H&E	Hematoxylin and eosin
HDL-C	High-density lipoprotein cholesterol
HK	Hexokinase
HO-1	Heme oxygenase-1
HSI	Hepatosomatic index
IFN-γ	Interferon gamma
IgH	Immunoglobulin heavy chain
IgM	Immunoglobulin M
Igλ1	Immunoglobulin lambda 1
IL	Interleukin (e.g., IL-1β, IL-6, IL-8, IL-10)
Keap1	Kelch-like ECH-associated protein 1
LDH	Lactate dehydrogenase
LDL-C	Low-density lipoprotein cholesterol
LPS	Lipase
LZM	Lysozyme
MAPK14	Mitogen-activated protein kinase 14
MDA	Malondialdehyde
Mn-SOD	Manganese superoxide dismutase
MT	Muscle thickness
NRAMP	Natural resistance-associated macrophage protein
Nrf2	Nuclear factor erythroid 2-related factor 2
PFK	Phosphofructokinase
PK	Pyruvate kinase
ROS	Reactive oxygen species
SD	Standard deviation
SGR	Specific growth rate
SOD	Superoxide dismutase
T-AOC	Total antioxidant capacity
TBA	Total bile acid
TC	Total cholesterol
TG	Triglyceride
TGF-β1	Transforming growth factor beta 1
TJ	Tight junction
TNF-α	Tumor necrosis factor alpha
